# International Conference on Technology and Innovation in Sports, Health and Wellbeing (TISHW): Vila Real, Portugal. 01-03 December 2016

**DOI:** 10.1186/s13102-017-0068-y

**Published:** 2017-02-24

**Authors:** Idoia Muñoz, Jokin Garatea, Jokin Garatea, Idoia Muñoz, Silvia Ala, Francisco Cardoso, Hugo Paredes, Margrit Gelautz, Florian Seitner, Christian Kapeller, Nicole Brosch, Zuzana Frydrychova, Iva Buresova, Katerina Bartosova, Sara Huteckova, Sara Huteckova, Iva Buresová, Katerina Bartosova, Zuzana Frydrychova, Marcelo Pires, Vítor Santos, Luís Almeida, Vitor Santos, Henrique Neiva, Mário Marques, Bruno Travassos, Daniel Marinho, Maria Helena Gil, Mário Cardoso Marques, Henrique Pereira Neiva, António Carlos Sousa, Daniel Almeida Marinho, António Carlos Sousa, Bruno Filipe Travassos, Maria Helena Gil, Henrique Pereira Neiva, Daniel Almeida Marinho, Mário Cardoso Marques, Tânia Rocha, Arsénio Reis, Hugo Paredes, João Barroso, Rimon Saffoury, Peter Blank, Julian Sessner, Benjamin Groh, Christine Martindale, Eva Dorschky, Joerg Franke, Bjoern Eskofier, Graça Barros, Filipe Melo, Raul Oliveira, Jorge Borges, Arsénio Reis, Vitor Santos, João Barroso, Arsénio Reis, Dennis Paulino, Hugo Paredes, Vitor Filipe, João Barroso, Arsénio Reis, Vitor Santos, Hugo Paredes, Vitor Filipe, João Barroso, João Ribeiro, Arsénio Reis, Elsa Justino, Vitor Santos, Arsénio Reis, João Barroso, Vasco Amorim, Vitor Filipe, Dennis Paulino, Arsénio Reis, Hugo Paredes, João Barroso

**Affiliations:** 1 GAIA, Association of Electronic and Information Technologies in the Basque Country, Bilbao, Spain; 2 GAIA, Association of Electronic and Information Technologies in the Basque Country, Bilbao, Spain; 3 Universidade de Trás-os-Montes e Alto Douro, Vila Real, Portugal; 4 INESC TEC and Universidade de Trás-os-Montes e Alto Douro, Vila Real, Portugal; 5 Vienna University of Technology, Vienna, Austria; 6 emotion3D GmbH, Vienna, Austria; 7 Department of Psychology, Masaryk University, Faculty of Arts, Brno, Czech Republic; 8 Department of Psychology, Masaryk University, Faculty of Arts, Brno, Czech Republic; 9 Universidade Nova de Lisboa, NOVA IMS, Lisboa, Portugal; 10 NOVA IMS, Lisboa, Portugal; 11 Department of Sport Sciences and CIDESD, University of Beira Interior, Covilhã, Portugal; 12 Department of Sport Sciences, University of Beira Interior and Research Centre in Sports Sciences, Health Sciences and Human Development, Covilhã, Portugal; 13 Department of Sport Sciences, University of Beira Interior, Research Centre in Sports Sciences, Health Sciences and Human Development, Covilhã, Portugal; 14 INESC TEC and University of Trás-os-Montes and Alto Douro, Vila Real, Portugal; 15 Digital Sports Group, Pattern Recognition Lab, Department of Computer Science, Friedrich-Alexander University Erlangen-Nuremberg (FAU), Erlangen, Germany; 16 Institute for Factory Automation and Production Systems, Friedrich-Alexander University Erlangen-Nuremberg (FAU), Erlangen, Germany; 17 Fisio Lógic Centro de Fisioterapia, Lisboa, Portugal; 18 Faculdade de Motricidade Humana, Universidade de Lisboa, Laboratório de Comportamento Motor, Estrada da Costa, 1499-002 Cruz Quebrada, Portugal; 19 Universidade de Trás-os-Montes e Alto Douro (UTAD), Vila Real, Portugal; 20 Universidade de Trás-os-Montes e Alto Douro (UTAD) and Instituto de Engenharia de Sistemas e Computadores, Tecnologia e Ciência (INESC TEC), Vila Real, Portugal; 21 Universidade Nova de Lisboa, NOVA IMS, Lisboa, Portugal; 22 Universidade de Trás-os-Montes e Alto Douro (UTAD) and Instituto de Engenharia de Sistemas e Computadores, Tecnologia e Ciência (INESC TEC), Vila Real, Portugal; 23 Universidade de Trás-os-Montes e Alto Douro (UTAD) and Instituto de Engenharia de Sistemas e Computadores, Tecnologia e Ciência (INESC TEC), Vila Real, Portugal; 24 Universidade Nova de Lisboa, NOVA IMS, Lisboa, Portugal; 25 Universidade de Trás-os-Montes e Alto Douro (UTAD), Vila Real, Portugal; 26 Universidade de Trás-os-Montes e Alto Douro (UTAD) and Instituto de Engenharia de Sistemas e Computadores, Tecnologia e Ciência (INESC TEC), Vila Real, Portugal; 27 Universidade Nova de Lisboa, NOVA IMS, Lisboa, Portugal; 28 INESC TEC and Universidade de Trás-os-Montes e Alto Douro, Vila Real, Portugal; 29 INESC TEC and University of Trás-os-Montes e Alto Douro, Vila Real, Portugal; 30 INESC TEC and University of Trás-os-Montes e Alto Douro, Vila Real, Portugal

## A1 ObesiTIC. Collaborative design for health improvement through physical activity and sports

### Idoia Muñoz, Jokin Garatea

#### GAIA, Association of Electronic and Information Technologies in the Basque Country, Bilbao, Spain

##### **Correspondence:** Idoia Muñoz (idoia@gaia.es)

**Introduction**

ObesiTIC is a project which aims to investigate innovative information and communication technologies resulting in a new ICT tool specifically designed for children and teenagers, in order to acquire healthy lifestyles, promoting physical activity and avoiding health and social problems associated with obesity and overweight. This is achieved through its co-design and validation with children and teens following a Living Lab approach through SPORTIS Living Lab, a European Network of Living Lab’s effective member.

**Objectives**

1. To develop an innovative solution that would enable health-related behaviour changes, increase motivation, promote physical activity and reduce prolonged sedentary time in users, thanks to persuasive and ubiquitous computing techniques.

2. To be validated by SPORTIS Living Lab. Following SPORTIS aim to involve society in the innovation process, ObesiTIC will be validated by end-users (children and teenagers) combined with the development of the application and final product, in order to suit and respect all the needs and aspects of the users’ requirements.

**Methods**

A Living Lab methodology is implemented:

1. Exploring and identifying potential end users
2. Co-design with end users
3. Testing through focus groups

**Expected Results**

1. Visibility, recognition and implication by the administration, user groups and SMEs.
2. Different cross-border and local activities, like the People Olympics Initiative, which is an international initiative for social innovation based on collective physical activity competitions between cities around the world.
3. Scale the project up to all ages and to different European countries contributing to the initiative, involving European SMEs to test and validate it.

## A2 Ocean Living Lab. An innovative structure for co-conception and experimentation in nautical sports

### Jokin Garatea, Idoia Muñoz

#### GAIA, Association of Electronic and Information Technologies in the Basque Country, Bilbao, Spain

##### **Correspondence:** Idoia Muñoz (idoia@gaia.es)

**Introduction**

Ocean Living Lab comprises a new level of collaborative endeavour, pooling energies, capabilities and methodologies to resolve common problems, to innovate around new uses and to test new equipment in nautical sports much more effectively.

Ocean LL intends to address the territorial challenges of promoting the marine/ aquatic sector and the competitiveness of these enterprises through open innovation, bringing together economic operators (water sector and technologies), user communities, R&D centers and universities to build a marine sector of excellence and an economic development engine.

**Objectives**

- To foster the practice of the greatest number of water-related activities by developing new and entirely safe usages in a preserved environment.
- To make the Basque coast a land of excellence for co-conception and experimentation in real conditions of new products and services in nautical sports by developing optimal conditions for participation by all kinds of users in terms of access, safety, progress and satisfaction.

**Methods**

A methodology comprised of six stages:

- Ideas
- Conception
- Building of prototypes
- Testing
- Evaluation
- Commercialization

**Expected Results**

- Mobilisation of industrial partners
- Promoting the surf and nautical industry in the territory
- Becoming the European reference center for the development of new products and services for the Ocean sliding sector and water activities.
- Divide the ""Time to Market" and “project risks" in 2.

## A3 e-Health usability and acceptance: A case study of the Portuguese citizens’ health portal

### Silvia Ala^1^, Francisco Cardoso^1^, Hugo Paredes^2^

#### ^1^Universidade de Trás-os-Montes e Alto Douro, Vila Real, Portugal; ^2^INESC TEC and Universidade de Trás-os-Montes e Alto Douro, Vila Real, Portugal

##### **Correspondence:** Hugo Paredes (hparedes@utad.pt)

**Introduction**

Nowadays, ICT can play a significant role as an administrative interface between citizens and the health services. However, the interaction between services and the users will only be effective if the platforms provided are known, accepted, and user-friendly. These dimensions are highlighted as a key to success for the application of ICT in health services by different technology-acceptance models (TAM, UTAUT, CAT).

**Objectives**

In this study, we aim to evaluate the level of information, acceptance, usability and usage intention of the Portuguese public health system portal (https://servicos.min-saude.pt/utente/).

**Methods**

This research was performed with one hundred participants (M = 30.5; SD = 1.23) from the north of Portugal. Initially, we collected information about the participants’ health system, and their level of information, frequency of use and influence within the portal. Later, participants interacted with the portal and rated it to determine the acceptability, ease of use and intention.

**Results**

The results reveal that 56% of individuals use only the National Health System for medical care; 62% of the participants did not know about the portal and 68% said that health professionals never informed them about the online service. 81% of the participants rated the portal as useful, 92% found it easy to use, and 90% intend to use it in the future.

**Conclusions**

Based on the results, we suggest the need for studies concerning the acceptance and use of technologies in the health domain and about the impact that these systems have on everyday life, especially for the elderly.

## A4 Stereo reconstruction of sports scenes: algorithms, applications, and challenges

### Margrit Gelautz^1^, Florian Seitner^2^, Christian Kapeller^1^, Nicole Brosch^1^

#### ^1^Vienna University of Technology, Vienna, Austria; ^2^emotion3D GmbH, Vienna, Austria

##### **Correspondence:** Margrit Gelautz (margrit.gelautz@tuwien.ac.at)

Stereo analysis allows us to reconstruct a dynamic 3D scene from two or more input videos taken from slightly displaced viewpoints. The computed 3D information (or depth map) forms the basis for a variety of applications such as novel view synthesis, virtual camera motion, or the combination of real and synthetic video content for augmented reality scenarios, which can greatly enhance the sports viewing experience.

Our goal is to review the stereo processing pipeline and stereo matching algorithms from the particular perspective of sports applications. While the available literature focuses mostly on coarse depth reconstructions of sports scenes (for example, for extracting soccer players from the background), we aim to generate high-quality depth maps that reveal the subtleties of the athlete’s pose and movement.

We provide an overview of the state-of-the-art computer vision algorithms for stereo analysis and discuss the special challenges that are posed to stereo matching by different types of sports scenarios. Examples of such challenges are fast and/or complex body movements, occlusions between team players, and unfavourable illumination or surface reflectance conditions.

We show results that were obtained by applying state-of-the-art stereo matching algorithms and semi-automatic 2D-to-3D conversion techniques to sports scenes. In addition, we provide suggestions for future stereo capture and processing systems that are tailored to the peculiarities of the sports domain and discuss potential applications.

## A5 Parental motivational climate – a risk factor contributing to overtraining syndrome in young elite athletes?

### Zuzana Frydrychova, Iva Buresova, Katerina Bartosova, Sara Huteckova

#### Masaryk University, Faculty of Arts, Department of Psychology, Brno, Czech Republic

##### **Correspondence:** Zuzana Frydrychova (frydrychova.zuzka@gmail.com)

Parents are the main influence on the overall psychosocial development of their child. Their influence is not limited to one specific environment (respectively a sports environment), but it is rather general. The aim of this study is to explore the theoretical bases regarding parental motivational climate as a risk factor in the development of overtraining syndrome in young elite athletes and to examine the topic on the Czech population. In the present study the following questionnaires were used: Profile of Mood States (POMS), The SFMS questionnaire, Sport Motivation Scale (SMS) and Parent-Initiated Motivational Climate Questionnaire-2 (PIMCQ-2). Preliminary results will be presented concerning the role of parental motivational climate in the development of overtraining syndrome in adolescent elite athletes. In the case of young elite athletes, the quality of the parent-child relationship is considered as a major predictor of motivation, enjoyment and stress level associated with sports activities. Whether parents create mastery motivational climate is usually connected with intrinsic motivation, better psychosocial and performance outcomes, and lower levels of anxiety in young athletes. Conversely, parental pressure and an ego-involving motivational climate are related to negative affect, higher levels of anxiety and extrinsic motivation. Perceived stress, anxiety and overload may contribute to the development of overtraining syndrome.

## A6 Personal traits of elite athletes in the context of overtraining syndrome in adolescence

### Sara Huteckova, Iva Buresová, Katerina Bartosova, Zuzana Frydrychova

#### Masaryk University, Faculty of Arts, Department of Psychology, Brno, Czech Republic

##### **Correspondence:** Sara Huteckova (sara.huteckova@gmail.com)

Regular intensive training associated with mental and physical effort can lead to overreaching in young elite athletes. The main purpose of this study is to map the theoretical bases to examine the role of selected personality traits in the context of overtraining syndrome in young elite athletes and to provide some preliminary results concerning possible relationships between personality traits and overtraining syndrome. To examine possible relationships between overtraining and selected personality traits the study uses the following methods: Neo Five-Factor Inventory (NEO-FFI), Profile of Mood States questionnaire (POMS), The SFMS questionnaire, Sport Motivation Scale (SMS). In relation to the lack of rest, insufficient regeneration, stress and load before and during competitions, the risk of overtraining syndrome increases. Chronic fatigue, underperformance, inability to compete, sleep disturbances and mood disorders can be considered as the most significant symptoms of the development of overtraining syndrome in young elite athletes. An athlete’s personality plays an important role in his or her sports career. Certain personality traits contribute to the achievement of positive results in sports. Nevertheless, the relationship between personality traits and sports performance can be reversed. Achieving a higher performance, experiencing success and the life changes associated with a sports career can also lead to changes in an elite athlete’s personality. Personality traits differ among athletes and the physically inactive population. Differences can also be identified between athletes engaged in individual and team sports.

## A7 Use of “Internet of Everything” technologies in sports

### Marcelo Pires, Vítor Santos

#### Universidade Nova de Lisboa, NOVA IMS, Lisboa, Portugal

##### **Correspondence:** Vítor Santos (vsantos@novaims.unl.pt)

**Introduction**

In an age where there are devices and applications for almost everything in the daily life of a modern society, it becomes important to assess their impact on the users’ lives, as well as their increasing development in an even more technological and connected future than the present time. One growing area is the use of these devices in sports.

Since the increasing use of these devices, the sports landscape has been changing, both in terms of monitoring the athletes' welfare, as well as in improving performances and results in training and competitions in which they participate. Sports Technology is in constant expansion and development, as we witness a greater involvement of science and technology in sports, more than ever before.

In the Big Data era, sports are also included t, because, there are increasingly large amounts of data collected that can be applied for analysis, thereby creating competitive advantages to be used either in real-time during a competition or during practice, preparation or recruitment.

Will the use of these devices extend further in the future or are we over-automating sports that want athletes to be the main focus?

**Objectives**

The main focus of this research is to study the technologies related to the “Internet of Everything” and the devices impacting sports. We will assess the role that these devices have nowadays and the role they will come to play in the future, evaluating their suitability in each sport. We will analyse the advantages and disadvantages of adopting these devices according to their suitability to different sports.

**Methods**

First, the research will focus on the use of technology in sports and on defining the initial requirements. Then, we will implement a suitability matrix with each sport and its main devices, presenting a ranking according to suitability. Later, we will design a questionnaire to assess the results of this matrix.

**Expected Results**

The results of this project will provide a better evaluation of the current situation of the use of technology in sports.

## A8 Information systems in gym sports

### Luís Almeida, Vitor Santos

#### NOVA IMS, Lisboa, Portugal

##### **Correspondence:** Luís Almeida (77luis.almeida@gmail.com)

**Introduction**

With the increase of available information about what is better for our health, the business of gyms has undergone considerable growth. Another area with an important development in the past few years has been technology. It is important to understand how connected these two subjects are. Our research has two main perspectives: the use of technology on an individual basis through smartphone apps, with the purpose to help athletes achieve their desired level; the use of technology on a collective level through the modernization of gym equipment to support and record the level of the athlete.

**Objectives**

The main objective of this work is to evaluate and understand the current situation of the applicability of information systems for gym sports. To achieve the proposed goal we will have two approaches: at the individual level, trying to understand the use of apps by athletes in their sports activities and, second, at the collective level trying to evaluate the level of technology embedded in the gym machines and how useful they are.

**Methods**

First, the research will focus on the use of technology in sports and on determining the initial requirements. After that, we will elaborate and apply a questionnaire about technology in gyms, and analyse the results, providing some conclusions and limitations.

**Expected Results**

The results of this project will provide a better evaluation of the current situation of technology in gym sports, the usedness of technology in sports gym in terms of usefulness and what is missing to make them more complete.

## A9 Aquatic fitness training programs to improve health-related parameters: advanced monitoring tools

### Henrique Neiva, Mário Marques, Bruno Travassos, Daniel Marinho

#### University of Beira Interior, Department of Sport Sciences and CIDESD, Covilhã, Portugal

##### **Correspondence:** Henrique Neiva (henriquepn@gmail.com)

**Introduction**

Aquatic physical training programs have increased in popularity over the years because of the several benefits in terms of physical fitness and health parameters. Cardiovascular and metabolic adaptations are the main evidence, but there is some information lacking in our comprehensive knowledge about them. In addition, these programs need to be regularly controlled, especially with regard to intensity, to cause enough adaptations and to maintain program adhesion.

**Objectives**

To examine the current research concerning the adaptations of healthy subjects to aquatic fitness activities and the monitoring techniques used to control these adaptations.

**Methods**

Several databases were searched for studies published from 2000 to 2016. Studies about water-based programs that analysed physiological and anthropometric changes in healthy subjects were included. Specific keywords were used and only studies written in English, published in a peer-reviewed journal, were considered.

**Expected Results**

Health-related parameters were improved after an aquatic fitness program of at least 8-weeks. Studies usually evaluated cardiovascular and metabolic adaptations (oxygen uptake and heart rate), muscular strength and endurance (isokinetic and repetitions to exhaustion), body composition (skinfold thickness, bioimpedance) and flexibility (sit-and-reach). On a regular basis, perceived exertion, heart rate, blood lactate, and oxygen uptake were used to monitor the intensity and improve the efficiency of the program. However, the emerging wide range of sensors may enable new evaluation possibilities and allow the practitioners to access timely feedback on their acute responses to improve their physical fitness. These wearables can be challenging and bring new highlights for aquatic fitness programs in a near future.

## A10 External heating garments used post-warm-up and effects in sports performance

### Maria Helena Gil, Mário Cardoso Marques, Henrique Pereira Neiva, António Carlos Sousa, Daniel Almeida Marinho

#### Department of Sport Sciences, University of Beira Interior and Research Centre in Sports Sciences, Health Sciences and Human Development, Covilhã, Portugal

##### **Correspondence:** Maria Helena Gil (maria.helena.gil@hotmail.com)

**Introduction**

There are several studies that have reported significant losses in body temperature and athletic performance after the transition between the warm-up period and sports competition. The passive temperature maintenance is one method used to minimize the reduction in body temperature, yet the literature remains unclear on this issue.

**Objectives**

This study aimed to present data concerning the benefits of using thermal garments in the transition period between warming-up/heating and sports events.

**Methods**

This work was based on articles indexed in several databases as ISI Web of Knowledge, PubMed and ScienceDirect. For further analysis the following keywords were included separately and/or combined: post warm-up, passive heating, external heating, garments of heating.

**Expected Results**

Passive heating involves the use of specific methods (i.e. thermal garments, survival jackets and heating pads) to attenuate heat loss. These are easily administered in order to maintain specific muscles temperature. An interval of 30 minutes leads to a decrease in muscle temperature (Tm) and core temperature (Tc). In turn, passive temperature maintenance during the interval reduces the decline in Tc, leading to an improvement in peak power as well as repeated sprint capacity. A 1°C reduction in Tm leads to a 3% reduction in muscle power of the legs and the increase of 1°C in Tm can improve 2-5% of the subsequent performance. In conclusion, the use of thermal garments during the transition phase between warming-up and the sports events can be of great importance in maintaining the temperature and in enhancing sports performance.

**Acknowledgements**

This work was supported by Project “NanoSTIMA: Macro-to-Nano Human Sensing: Towards Integrated Multimodal Health Monitoring and Analytics/NORTE-01-0145-FEDER-000016" financed by the North Portugal Regional Operational Programme (NORTE 2020), under the PORTUGAL 2020 Partnership Agreement, and through the European Regional Development Fund (ERDF).

## A11 Wearable technologies in recreational team sports: a brief review

### António Carlos Sousa, Bruno Filipe Travassos, Maria Helena Gil, Henrique Pereira Neiva, Daniel Almeida Marinho, Mário Cardoso Marques

#### Department of Sport Sciences, University of Beira Interior, Research Centre in Sports Sciences, Health Sciences and Human Development, Covilhã, Portugal

##### **Correspondence:** António Carlos Sousa (antonio_carlossousa@hotmail.com)

**Introduction**

Physical inactivity is considered a major health problem and is associated with diseases such as hypertension, obesity and hyperglycemia, reducing cardiovascular and respiratory functions. Regular exercises are highly recommended to prevent and treat these diseases. The main barriers to engaging in physical activity include high cost, poor access to facilities, lack of time and motivation. In this context recreational team sports and leisure assumed a significant role in battling sedentary modern lifestyle. Unfortunately, the literature is scarce about the use of wearable technology (WT) in recreational team sports, which help monitor the effects of such activities. **Objective**: The purpose was to examine the use of WT on recreational team sports and provide perspectives for future research on inactive/sedentary subjects.

**Methods**

Four electronic databases (ISI Web of Knowledge, PubMed, SPORTDiscus and Web of Science) were searched for original research articles. A search was performed to cover the areas of recreational team sports and recreational small-sided games using the following key terms, either by itself or in combination: recreational small sided games, team sports, portable sensors, technology.

**Results**

The most monitored variables to provide accurate assessments of physical and health condition were: heart rate, maximal oxygen uptake, blood pressure and blood lactate concentration. In brief, WT can actually be an important tool/instrument to monitor physical condition in individuals participating in various recreational team sports. Therefore, more studies using other portable devices are needed to get more feedback on the physical condition and health of the subjects.

**Acknowledgements**

This work was supported by Project “NanoSTIMA: Macro-to-Nano Human Sensing: Towards Integrated Multimodal Health Monitoring and Analytics/NORTE-01-0145-FEDER-000016" financed by the North Portugal Regional Operational Programme (NORTE 2020), under the PORTUGAL 2020 Partnership Agreement, and through the European Regional Development Fund (ERDF).

## A12 BuyMe: Didactic game for the cognitive training of Children and the Elderly

### Tânia Rocha, Arsénio Reis, Hugo Paredes, João Barroso

#### INESC TEC and University of Trás-os-Montes and Alto Douro, Vila Real, Portugal

##### **Correspondence:** Tânia Rocha (trocha@utad.pt)

**Introduction**

A didactic game to cognitively stimulate children and the elderly is presented. For that, the game interface presented a series of images where the participants had to memorize a shopping list (with products and quantities) and then had to follow the right path to collect the products. The level of difficulty increased when they had to collect the products and the right quantities.

**Objectives**

With this game, we aim to cognitively stimulate children and the elderly through memory and logic as they have to memorize products presented in a list, verify the right quantities of the products and also choose the right path to conclude the game [1] [2]. Also, it is designed to be a didactic game as it teaches about products, prices and product sections.

**Methods**

After developing the game, we carried out a case study where we registered usability variables, such as: effectiveness (the capacity to accomplish the proposed task), efficiency (errors, difficulties in the interaction) and satisfaction (comfort and acceptance when performing the task).

**Expected results**

The children had better performances than the elderly regarding effectiveness, efficiency and satisfaction. We believe the user interface for the elderly needs to be improved because the game must be played with the mouse input device.

**Acknowledgements**

This work was supported by Project “NanoSTIMA: Macro-to-Nano Human Sensing: Towards Integrated Multimodal Health Monitoring and Analytics/NORTE-01-0145-FEDER-000016" financed by the North Portugal Regional Operational Programme (NORTE 2020), under the PORTUGAL 2020 Partnership Agreement, and through the European Regional Development Fund (ERDF).

**References**

[1] Marcelino, I., Barroso, J., Bulas Cruz, J. & Pereira., A. (2010). Elder Care Architecture–a physical and social approach. In C. Stephanidis & M. Antona (Eds.), International Journal on Advances in Life Sciences, Volume 2, Number 1 & 2. 2010, 61.2 (29) pp.9.

[2] Rocha, T., Paredes, H., Barroso, J. & Bessa. M. (2016) SAMi: an accessible Web application solution for video search for people with intellectual disabilities. 15th International Conference on Computers Helping People with Special Needs, July 13-15, 2016, University of Linz, Austria. In Miesenberger, K., Bühler, C., Penaz, P. (Eds.) Computers Helping People with Special Needs Volume 9759 of the series Lecture Notes in Computer Science pp. 310-316.

## A13 Blind path obstacle detector using a smartphone camera and a line laser emitter

### Rimon Saffoury^1^, Peter Blank^1^, Julian Sessner^2^, Benjamin Groh^1^, Christine Martindale^1^, Eva Dorschky^1^, Joerg Franke^2^, Bjoern Eskofier^1^

#### ^1^Digital Sports Group, Pattern Recognition Lab, Department of Computer Science, Friedrich-Alexander University Erlangen-Nuremberg (FAU), Erlangen, Germany; ^2^Institute for Factory Automation and Production Systems, Friedrich-Alexander University Erlangen-Nuremberg (FAU), Erlangen, Germany

##### **Correspondence:** Rimon Saffoury (Rimon.saffoury@fau.de)

Visually impaired people find navigating within unfamiliar environments challenging. Many smart systems have been proposed to help blind people in these difficult, often dangerous, situations. However, some of them are uncomfortable, difficult to obtain or simply too expensive. In this paper, a low-cost wearable system for visually-impaired people was implemented, which allows them to detect and locate obstacles in their location. The proposed system consists of two main hardware components, a laser pointer ($12) and an Android smartphone, making our system relatively cheap and accessible. The collision avoidance algorithm uses image processing to measure distances to objects in the environment. This is based on laser light triangulation. This obstacle detection is enhanced by edge detection within the captured image. An additional feature of the system is to recognize and warn the user when stairs are present in the camera’s field of vision. Obstacles are brought to the user’s attention using an acoustic signal. Our system was shown to be robust, with only a 5% false alarm rate and a sensitivity of 90% for 1 cm wide obstacles.

## A14 Dual Task effects on posture and gait control of elderly people

### Graça Barros^1^, Filipe Melo^2^, Raul Oliveira^2^

#### ^1^Fisio Lógic Centro de Fisioterapia, Lisboa, Portugal; ^2^Faculdade de Motricidade Humana, Universidade de Lisboa, Laboratório de Comportamento Motor, Estrada da Costa, 1499-002, Cruz Quebrada, Portugal

##### **Correspondence:** Filipe Melo (fmelo@fmh.ulisboa.pt)

**Introduction**

Daily motor tasks, mostly composed of dual task conditions (or multitasking), involve the interaction of perceptual-motor and cognitive neurophysiologic processes able to influence postural control. Due to its inherent modifications, ageing places constraints on many of these processes, which are crucial to the recovery or maintenance of good balance.

**Objective**

The main goal is to determine the effects of dual task accomplishment on postural static and dynamic balance on elderly people, and to analyse the inherent variation on the different temporal and space parameters as a consequence of performing these different tasks.

**Methods**

The research involved an observational cross study with a single moment evaluation. Our sample included 36 subjects, (9 men and 27 woman, 73 ± 5,7 years old). Kinematic data were achieved using an APDM® Mobility Lab system during two tests – ISWAY and ITUG. The results allowed us to identify the influence of the different task conditions (single, dual and multitask) on static (standing position) and dynamic balance (stand to sit, straight walking, turning and turn to sit) in elderly subjects.

**Results**

The results suggest that cognitive processes are a main cause of increased variability in postural sway and gait. In the activities analysed, such as sit to stand, turning and turn to sit, the simple motor task condition and the perceptive information related becomes the most important focus of attention, while any cognitive task becomes secondary.

## A15 Using NFC to manage users and infrastructures

### Jorge Borges^1^, Arsénio Reis^2^, Vitor Santos^3^, João Barroso^2^

#### ^1^Universidade de Trás-os-Montes e Alto Douro (UTAD), Vila Real, Portugal; ^2^Universidade de Trás-os-Montes e Alto Douro (UTAD) and Instituto de Engenharia de Sistemas e Computadores, Tecnologia e Ciência (INESC TEC), Vila Real, Portugal; ^3^Universidade Nova de Lisboa, NOVA IMS, Lisboa, Portugal

##### **Correspondence:** Jorge Borges (jborges@utad.pt)

**Introduction**

NFC stands for Near Field Communications and is a set of short-range wireless technologies, typically requiring a separation of 10 cm or less. NFC protocols establish a generally-supported standard, in contrast with other close-range communication proprietary technologies currently used for specific applications, such as, ID cards, access control and payment readers. NFC devices can operate in different modes: NFC card emulation; NFC reader and writer; NFC peer-to-peer communication. The latter mode is particular interesting, as it allows the exchange of data between two devices in a short-range context. Also, when one of the devices has internet connectivity, the other can exchange data with online services. Using this feature, associated with a specific developed app, a mobile NFC enabled device might become a mobile platform to acquire, manage and present information to a user, by reading data from closed range devices, have it processed through an online service and present the relevant feedback to the user.

**Objectives**

The main objective is to use NFC-enabled portable devices, with specific apps, to identify users and register their activities. Specific usages are: user identification, access control and infrastructure management.

**Methods**

Specific projects will be developed, regarding specific NFC usage scenarios: mobile phone usage for student identification in a classroom; tags and mobile phone usage for activity tracking in a gym; application to analyse the user activity in a gym and provide infrastructure management options. Special care will also be given to the NFC usage to improve user accessibility [1].

**Expected Results**

We expect to better understand how the NFC standard can enable the development of user-centred applications, previously restricted by proprietary communications protocols.

**Acknowledgements**

This work was supported by Project ""ENSINO@IES”, Ref. T469788954-00025468, financed by SAMA2020/2016: Sistema de Apoio à Modernização e Capacitação da Administração Pública.

**References**

[1] Reis, A., Barroso, J., & Gonçalves, R. (2013). Supporting Accessibility in Higher Education Information Systems. In C. Stephanidis & M. Antona (Eds.), Universal Access in Human-Computer Interaction. Applications and Services for Quality of Life: 7th International Conference, UAHCI 2013, Held as Part of HCI International 2013, Las Vegas, NV, USA, July 21-26, 2013, Proceedings, Part III (pp. 250-255). Berlin, Heidelberg: Springer Berlin Heidelberg.

## A16 Using cloud image processing services

### Arsénio Reis, Dennis Paulino, Hugo Paredes, Vitor Filipe, João Barroso

#### Universidade de Trás-os-Montes e Alto Douro (UTAD) and Instituto de Engenharia de Sistemas e Computadores, Tecnologia e Ciência (INESC TEC), Vila Real, Portugal

##### **Correspondence:** Arsénio Reis (ars@utad.pt)

**Introduction**

Currently, mobile vision technology is able to use data processing services in the cloud, allowing thinner devices, such as smartphones, to collect images and videos and have them processed on the cloud, using powerful algorithms and benefiting from machine learning, deep learning and several other developments related with artificial intelligence.

**Objectives**

This study has the objective of assessing the current status of the image-related services and their usability as building blocks in specific user applications.

**Methods**

In order to do so, several apps will be developed to test the image-processing cloud services in three different contexts and to evaluate the suitability of the services. The contexts will be: (1) assistive technology for blind people, identifying situations to improve their autonomy; (2) outdoor sports, such as bicycle or motorized sports, where augmented reality with image processing could, for example, draw the best route on a track; (3) in the workplace, such as, in an assembly line to detect errors in assembling an object, with image recognition.

**Expected results**

The expected results would indicate how suitable the current image processing cloud services are in the proposed contexts.

**Acknowledgements**

This work was supported by Project “NanoSTIMA: Macro-to-Nano Human Sensing: Towards Integrated Multimodal Health Monitoring and Analytics/NORTE-01-0145-FEDER-000016" financed by the North Portugal Regional Operational Programme (NORTE 2020), under the PORTUGAL 2020 Partnership Agreement, and through the European Regional Development Fund (ERDF).

## A17 Automating user supervision at the gym

### Arsénio Reis^1^, Vitor Santos^2^, Hugo Paredes^1^, Vitor Filipe^1^, João Barroso^1^

#### ^1^Universidade de Trás-os-Montes e Alto Douro (UTAD) and Instituto de Engenharia de Sistemas e Computadores, Tecnologia e Ciência (INESC TEC), Vila Real, Portugal; ^2^Universidade Nova de Lisboa, NOVA IMS, Lisboa, Portugal

##### **Correspondence:** Arsénio Reis (ars@utad.pt)

**Introduction**

In most gyms, users execute their fitness activities with mild supervision from a trainer that prescribes and periodically adjusts and instructs the user. Most users would benefit from a closer supervision.

**Objectives**

The objective is to introduce a platform that collects the user’s activities in the gym and processes that information according to his or her saved personal information, e.g., physiology, health condition, exercise prescription. The platform will mediate the relationship between the user and his trainer by providing information to the user and to the trainer about the progresses. By processing the user’s activity information, the platform should identify trends (divergence/convergence) and alert the trainer.

**Methods**

We expect to develop an incremental project, by starting with user monitoring and adding artificial intelligence capabilities to process and identify patterns in the user’s data. Particular care will be given to specific scenarios such as stroke rehabilitation [1].

**Expected Results**

We expect to promote a closer supervision of the user by the system, maximizing the trainer’s activity and lowering the costs of supervising the users.

**References**

[1] Reis, A., Lains, J., Paredes, H., Filipe, V., Abrantes, C., Ferreira, F., Mendes, R., Amorim, P., Barroso, J. (2016). Developing a System for Post-Stroke Rehabilitation: An Exergames Approach. In M. Antona & C. Stephanidis (Eds.), Universal Access in Human-Computer Interaction. Users and Context Diversity: 10th International Conference, UAHCI 2016, Held as Part of HCI International 2016, Toronto, ON, Canada, July 17-22, 2016, Proceedings, Part III (pp. 403-413). Cham: Springer International Publishing.

## A18 ActiveGym, inspiring educational success and healthy lifestyles at UTAD

### João Ribeiro^1^, Arsénio Reis^2^, Elsa Justino^1^

#### ^1^Universidade de Trás-os-Montes e Alto Douro (UTAD), Vila Real, Portugal; ^2^Universidade de Trás-os-Montes e Alto Douro (UTAD) and Instituto de Engenharia de Sistemas e Computadores, Tecnologia e Ciência (INESC TEC), Vila Real, Portugal

##### **Correspondence:** João Ribeiro (joaoribeiro@utad.pt)

**Introduction**

Throughout history, sports have played an important role in all human societies and are an essential element for a full personal development. In higher education, sports practice is used as a strategy to inspire educational success and healthy lifestyles and should be promoted in its different forms, e.g., competitive sport, physical activity or game play.

**Objectives**

It is the aim of SASUTAD to promote sports, cultural and social participation, within the academic community (students, staff, faculty and alumni), by providing conditions for democratic access to these practices in an educational environment, characterized by its excellence, health and openness to the community.

**Methods**

SASUTAD intends to increase, improve and diversify the sports practice offers, through the adoption of a rational and innovative management, in close cooperation with the students’ association and with the education and sports department, as well as with other relevant entities. The development of the sports services takes into account the trends in performance sports and also those of school sports, recreation and leisure, tourism sports and especially the needs and interests of the general population within a healthy lifestyle perspective. These goals are carried out through the “UTAD – Active Academy” concept and ACTIVEGYM project, which provide various sports programs, e.g., bodybuilding, fitness, rhythms and other gym activities, team sports and individual sports, combat sports, adventure, leisure and recreation.

**Expected Results**

We expect to support the cultural and sports development of the UTAD academy, in close cooperation with the AAUTAD, and contribute to the success of the UTAD’s students, as well as to provide a healthier lifestyle option to the general population.

## A19 A game to improve memory in elderly

### Vitor Santos^1^, Arsénio Reis^2^, João Barroso^2^

#### ^1^Universidade Nova de Lisboa, NOVA IMS, Lisboa, Portugal; ^2^INESC TEC and Universidade de Trás-os-Montes e Alto Douro, Vila Real, Portugal

##### **Correspondence:** Vitor Santos (vsantos@novaims.unl.pt)

**Introduction**

Throughout life there are several cognitive processes that deteriorate. One is the memory function that allows storage of information and whose purpose is to help other brain functions, as exemplified by the recognition and recall processes. Forgetfulness is connected to the experience, time, capacity to order, and to the location of the information recalled. Research has shown there is a decline in memory during aging and stresses that certain memory deficits are part of a healthy aging process.

**Objectives**

The main objective of this work is to develop an interactive and entertaining software that could help the elderly to maintain their cognitive abilities. Thus, the final product will be a mix of game and personal agenda where the elderly will be challenged to both incorporate in the agenda activities that encourage them to remain youthful, as well as to store items related to their agenda or to produce memories.

**Methods**

We will use Design Science Research methodology to determine the initial requirements and the software architecture. The development will be done following an agile methodology.

**Expected results**

The results of this project allow the specification of an innovative daily life digital tool that could be used by elderly people to maintain their cognitive abilities. It Is also expected to create a new community with people that have the same interests and availability to get together.

**Acknowledgements**

This work was supported by Project “NanoSTIMA: Macro-to-Nano Human Sensing: Towards Integrated Multimodal Health Monitoring and Analytics/NORTE-01-0145-FEDER-000016" financed by the North Portugal Regional Operational Programme (NORTE 2020), under the PORTUGAL 2020 Partnership Agreement, and through the European Regional Development Fund (ERDF).

## A20 Combined technologies to improve air-pistol shooting technique

### Vasco Amorim, Vitor Filipe

#### INESC TEC and University of Trás-os-Montes e Alto Douro, Vila Real, Portugal

##### **Correspondence:** Vasco Amorim (vasco.amorim@inesctec.pt)

**Introduction**

The typical approach to air-pistol shooting technique consists of three elements: aiming, weapon holding and firing (pulling the trigger). Currently, these technologies are used occasionally in training contexts, but not combined, due to complexity of measurements and cost of individual setup. The training of fine motor skills for novice athletes can be improved by the early detection of technique flaws.

**Objectives**

The main goal of this study is to combine optic-electronic monitoring to assist coaches in training design. The secondary goal is to enable the identification of weak points in both the structure and organization of training and to appropriately adjust the training process with three different technologies combined.

**Methods**

To accomplish this, image processing from postural video analysis will be combined with optical-electronic monitoring (from SCATT) of each pistol shot and combined with breath analysis (from BioHarness device). The athletes’ breath detection devices and video analysis will provide clues to improve the shot plan procedure.

**Expected Results**

We expect that this experimental setup could provide a technological enhancement of air-pistol shooting coaching and improvement in performance.

## A21 Usage of mobile devices for monitoring and encouraging active life

### Dennis Paulino, Arsénio Reis, Hugo Paredes, João Barroso

#### INESC TEC and University of Trás-os-Montes e Alto Douro, Vila Real, Portugal

##### **Correspondence:** Dennis Paulino (dennis.l.paulino@inesctec.pt)

**Introduction**

Currently, mobile technology has the possibility to use many sensors for collecting health data from users such as heart rate, distance walked and even altitude. This technology, can be used for monitoring and promoting an active life for users.

**Objectives**

This study has the objective of evaluating the current technologies involved in collecting health data, and developing an app for mobile devices, to monitor users’ health status and encourage them, to maintain an active life.

**Methods**

To accomplish this purpose, an app for mobile devices will be developed , collecting data from heart rate, GPS signal, pedometer and altimeter (with altitude we can calculate inclination). Then we evaluate the user´s usability in different contexts. The contexts will be: (1) user exercising outdoor (2); user exercising indoor; (3) user performing daily activities.

**Expected Results**

The expected results will indicate if the developed application will be usable.

